# Ring chromosome 15 – cytogenetics and mapping arrays: a case report and review of the literature

**DOI:** 10.1186/s13256-018-1879-5

**Published:** 2018-11-16

**Authors:** César Paz-y-Miño, Jaime Guevara-Aguirre, Ariane Paz-y-Miño, Francesca Velarde, Isaac Armendáriz-Castillo, Verónica Yumiceba, Jesús María Hernández, Juan Luis García, Paola E. Leone

**Affiliations:** 1 Centro de Investigación Genética y Genómica, Facultad de Ciencias de la Salud Eugenio Espejo, Universidad UTE, Quito, Ecuador; 2 Facultad de Ciencias de la Salud, Universidad San Francisco de Quito, Quito, Ecuador; 3 Institute of Endocrinology, Metabolism, and Reproduction, Quito, Ecuador; 4 Unidad de Investigación en Biomedicina, Zurita & Zurita Laboratorios, Quito, Ecuador; 5 Servicio de Hematología, Hospital Universitario de Salamanca, Universidad de Salamanca, Salamanca, Spain; 6 Molecular Medicine Unit, Department of Medicine, Biomedical Research Institute of Salamanca (IBSAL), Salamanca, Spain; 7 Institute of Molecular and Cellular Biology of Cancer (IBMCC), University of Salamanca-SACYL-CSIC, Salamanca, Spain

**Keywords:** Ring 15, Mapping arrays, Cytogenetics, Ring review

## Abstract

**Background:**

Ring chromosome 15 has been associated in previous studies with different clinical characteristic such as cardiac problems, digit and musculoskeletal abnormalities, and mental and motor problems among others. Only 97 clinical cases of ring chromosome 15 syndrome have been reported since 1966 and a common phenotype for these patients has not been established.

**Case presentation:**

The present case report describes a 15-month-old girl from the Amazon region of Ecuador, of Mestizo ancestry, who after cytogenetic tests showed a 46,XX,r(15) karyotype in more than 70% of metaphases observed. Her parents were healthy and non-related. The pregnancy was complicated and was positive for intrauterine growth retardation. Her birth weight was 1950 g, her length was 43.5 cm, and she had a head circumference of 29.3. In addition to postnatal growth delay, she had scant frontal hair, small eyes, hypertelorism, low-set of ears, flattened nasal bridge, anteverted nostrils, down-turned mouth, three café au lait spots, and delayed dentition.

**Conclusions:**

Despite the frequency of some phenotypes expressed in the different clinical cases reviewed and the present case, a common phenotype for patients with ring 15 could not be determined and it is restricted to the region of the chromosome lost during the ring formation.

## Background

Rings are aberrant structures that arise from random events during cell replication, which yield a circular chromosome [1]. They have been identified for all human chromosomes [2]. Ring formation often results from terminal breaks in both chromosome arms, followed by a fusion event of the broken ends [1, 3]. As a result, genetic material may or may not be lost, leading to different types of rings [2]. Rings also occur due to the fusion of subtelomeric sequences like telomere–telomere fusion. This fusion might lead to complete ring formation with no significant loss of genetic material, as seen in patients with normal phenotype [1].

Due to the circular nature of the affected chromosomes, they tend to be unstable during cell division [4]. Sister chromatid exchanges due to cell mistakes during mitosis can result in chromosomal abnormalities that produce dicentric rings, interlocked rings, or ring loss leading to monosomy or mosaicism [4, 5]. Furthermore, transmission of unstable rings can lead to *de novo* ring formations in the next generation [2, 3, 6].

Ring chromosome 15 has been previously described in the literature. The first case reported [7], described a patient with café au lait macules, strabismus, and diminished mental and motor development. Further clinical features ranged from cardiac problems to digit and musculoskeletal abnormalities. Dysmorphic features, such as growth retardation, triangular faces, eye abnormalities, and developmental abnormalities are common features defined in ring 15 syndrome, which in this case report is abbreviated to r(15) syndrome [8, 9]. Since the first case report of a patient with ring chromosome 15 syndrome in 1966, only 97 clinical cases have been published in the literature to the date. In addition, it has been suggested that the clinical phenotype is correlated with the amount of deletions and genomic imbalances during the ring formation and the instability of the latter [1, 5, 10, 11].

This report describes the traits, demographic details, cytogenetic analysis, molecular diagnosis, and clinical manifestations of a baby girl with ring chromosome 15 and compares this case with other cases previously reported in the literature. The aim is to identify a common phenotype of ring chromosome 15 syndrome and improve our understanding of this genetic disorder.

## Case presentation

Our patient came from Coca city in the Amazon region of Ecuador; according to the ancestry profile of the Ecuadorian population [12] she is a Mestizo: 63.1% Native American, 30.3% European, and 6.6% African ancestry. The first contact with our patient was in April 2016 at 11-months old, she was the second child of non-consanguineous healthy parents. Her father was 32-years old and her mother was 31 at the time of birth. Our patient was born at 38 weeks of gestation from a complicated pregnancy. Intrauterine growth retardation, hypermature placenta, and low amniotic fluid were detected during the fifth month of pregnancy. The infant was delivered by cesarean C-section. Her birth weight was 1950 g (<third centile), her length was 43.5 cm (<third centile), and she had a head circumference (HC) of 29.3 (<third centile). The Appearance, Pulse, Grimace, Activity, and Respiration (APGAR) scores were 6–8. She required gastric probing, phototherapy, and thermo-cradle care for 10 days after birth.

At 15-months old after a physical examination, she showed a weight of 6000 g (<third centile), length of 63 cm (<third centile), and HC of 40 cm (third centile). In addition to postnatal growth delay, she had scant frontal hair, small eyes, hypertelorism, low-set of ears, flattened nasal bridge, anteverted nostrils, down-turned mouth, three café au lait spots, and delayed dentition. Developmentally, she showed normal psychomotor progress. She held up her head at 3 months, sat down at 6 months, and walked with support at 15 months. Bone age was determined by bone densitometry.

**Fig. 1:**
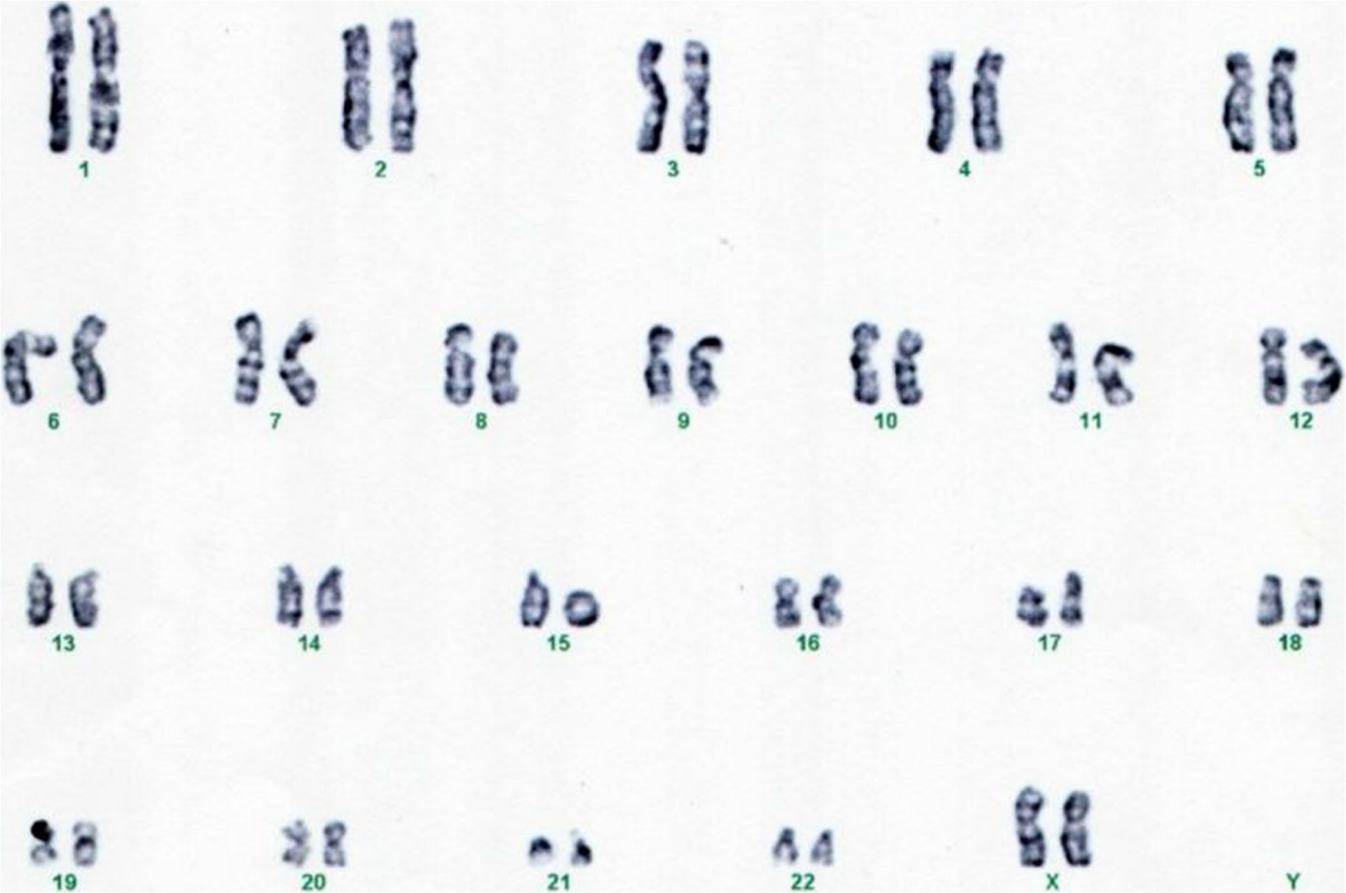
Karyotype of the patient from the present study showing a 46,XX,r(15)

A Giemsa trypsin banding (GTG) technique performed on our patient, at 450-band resolution, showed 73 out of 100 metaphases with a 46,XX,r(15) karyotype (Fig. 1). The parental karyotypes were normal. In addition, 750 ng of patient’s deoxyribonucleic acid (DNA) was used for hybridization in the Affymetrix 750K Array (Affymetrix, Santa Clara, CA, USA). The arrays were processed in the Fluidics Affymetrix 450 Station and scanned with GeneChip™ 3000 (Affymetrix, Santa Clara, CA, USA).

The arrays analysis revealed two copy number loss in the r(15)(q26.2q26.3) chromosomal region of 5,927,199 kbp, encompassing genes *NR2F2* to *OR4F13P*, and in the 15q26.3 chromosomal region of 3,459,976 kbp, encompassing genes *FAM169B* to *OR4F13P* (Table 1). The analysis also revealed additional abnormalities in chromosomes 14 and X. In chromosome 14, our patient had a three-copy gain of genetic information at 369,716 kbp in the 14q32.33 chromosomal region, including genes *KIAA0125, ADAM6*. While on chromosome X, there was a copy number loss of the genes *CT45A1* to *CT45A6* in the 119,101kbp region of Xq26.3 and a three-copy number gain of the genes spindlin family member 4 (*SPIN4*) and *LOC92249* in the 347,978kbp region of Xq11.1 (Table 2).

**Table 1:** Complete panel of ring 15 chromosome affected genes in the present case

Chromosomic region	Base pairs	Type	Number of copies	Genes
15q26.3	98,969,064-102,429,040	Loss	1	*FAM169B, IGF1R, MIR4714, PGPEP1L, SYNM, TTC23, LRRC28, HSP90B2P, MEF2A, LYSMD4, DNM1P46, ADAMTS17, SPATA41, CERS3, PRKXP1, LINS, ASB7, ALDH1A3, LRRK1, CHSY1, VIMP, SNRPA1, PCSK6, LOC100507472, TM2D3, TARSL2, OR4F6, OR4F15, OR4F13P*
15q26.2q26.3	96,501,841-102,429,040	Loss Mosaic	1.4	*NR2F2-AS1, NR2F2, MIR1469, SPATA8-AS1, SPATA8, LINC00923, ARRDC4, FAM169B, IGF1R, MIR4714, PGPEP1L, SYNM, TTC23, LRRC28, HSP90B2P, MEF2A, LYSMD4, DNM1P46, ADAMTS17, SPATA41, CERS3, PRKXP1, LINS, ASB7, ALDH1A3, LRRK1, CHSY1, VIMP, SNRPA1, PCSK6, LOC100507472, TM2D3, TARSL2, OR4F6, OR4F15, OR4F13P*

**Table 2:** Genetic abnormalities in other chromosomes in the present case

Chromosomic region	Base pairs	Type	Number of copies	Genes
14q32.33	106,342,949-106,712,665	Gain	3	*KIAA0125, ADAM6*
Xq26.3	134,854,915-134,974,016	Loss	1	*CT45A1, CT45A2, CT45A4, CT45A3, CT45A5, CT45A6*
Xq11.1	62,490,655-62,838,633	Gain	3	*SPIN4, LOC92249*

As a common phenotype for r(15) syndrome is not clearly described, we compared our patient case with 97 clinical cases of ring chromosome 15 found in the literature. In all 98 cases the average age at diagnosis was 10.85 years in females and 12.21 in males. The average maternal and paternal age at the time of birth ranged between 20 and 42 years (29.73%) and 22–46 (31.59%) years, respectively. Furthermore, the average weight and height for patients was 2252 g and 43.94 cm for females and 2345 g and 46.29 cm for males.

Among all phenotypic features, the following six were the most common in all 98 cases: growth retardation (76.53%), microcephaly (39.8%), clinodactyly (33.67%), triangular faces (32.65%), brachymesophalangy (32.65%), and low weight (42.86%). Other abnormalities for instance café au lait macules (25.51%), and psychological and behavioral abnormalities, such as developmental delay (27.55%), mental deficit (40.82%), and language deficit (27.55%) were also present (Table 3).

**Table 3:** Complete panel of clinical manifestations in ring 15 chromosome patients (*N* = 97)

Clinical features	Current case	Clinical cases	Total	%	Reference
Weight and height					
Weight (< 2100 g)	+	41	42	42.86	[4, 17, 22–39]
Height (< 46 cm)	+	24	25	25.51	[4, 7, 8, 17, 22–25, 29, 30, 38, 40–48]
Craniofacial					
Microcephaly	+	38	39	39.80	[4, 6, 8, 10, 15, 23–25, 29, 33–35, 37–39, 42, 44, 46, 47, 49–58]
Brachycephaly	–	5	5	5.10	[4, 10, 45, 50]
Hypertelorism	+	17	18	18.37	[6, 8, 16, 17, 24–26, 41, 42, 50, 51, 55, 59–62]
Frontal bossing	–	8	8	8.16	[4, 6, 10, 25, 28, 42, 51, 63]
Sparse temporal hair	+	5	6	6.12	[32, 35, 51, 52, 63, 64]
Short palpebral fissure	–	6	6	6.12	[17, 23, 32, 53, 54, 65]
Low and sharp tapering ears	+	16	17	17.35	[6, 7, 17, 23, 25, 29, 32, 33, 36, 42, 43, 53, 59, 61, 66, 67]
Triangular face	–	32	32	32.65	[8, 15, 24, 25, 27–30, 32–34, 38, 40, 43, 44, 46, 48, 50, 51, 54, 55, 57, 58, 66–68]
Broad nasal bridge	+	21	22	22.45	[8, 15, 18, 23, 24, 32–34, 36, 38, 44, 49, 63, 65, 67, 68]
Small nose	–	7	7	7.14	[8, 28, 32, 33, 44, 63]
Short philtrum	–	10	10	10.20	[6, 15, 25, 28, 35, 39, 48, 51, 56, 63]
Mouth with down-turned corners	+	8	9	9.18	[25, 32, 33, 43, 48, 54, 55, 69]
Hypoplasia of labia minora	–	5	5	5.10	[32, 34, 38, 44, 70]
High-arched palate	–	21	21	21.43	[4, 6, 8, 13, 17, 24–26, 30–32, 38, 44, 52–54, 59, 65, 66]
Epicanthal folds	–	9	9	9.18	[15, 34, 37, 39, 48, 52, 53, 63, 65]
Micrognathia	–	15	15	15.31	[15, 24, 25, 48, 51–55, 57, 63, 67, 68, 71]
Retrognathia	–	10	10	10.20	[4, 6, 8, 17, 34, 44, 45, 51, 70]
Strabismus	–	7	7	7.14	[18, 26, 29, 32, 54, 56, 66]
Musculoskeletal					
Delayed bone age	–	21	21	21.43	[6, 18, 22, 24, 30, 34, 37, 38, 43, 45–47, 53, 60, 62, 64, 72, 73]
Short neck	–	9	9	9.18	[8, 24, 32, 44, 48, 52]
Scoliosis	–	6	6	6.12	[4, 24, 38, 39, 41, 44]
Skin					
Abnormal simian crease	–	5	5	5.10	[32, 57, 59, 64, 65]
Café au lait macules	+	24	25	25.51	[4, 6, 15, 22, 28–30, 32, 37–39, 41, 46, 50, 51, 53, 54, 56, 59, 64, 66, 69, 74]
Cardiac					
Patent ductus arteriosus	–	5	5	5.10	[32, 33, 42, 43, 71]
Atrial septal defect	–	6	6	6.12	[6, 15, 18, 32, 33]
Ventricular septal defect	–	5	5	5.10	[15, 22, 33, 42]
Diaphragmatic hernia	–	7	7	7.14	[25, 33, 36, 47, 67]
Shield chest	–	5	5	5.10	[25, 42, 52, 68]
Hypoplasia of the mammary glands	–	6	6	6.12	[52]
Limbs					
Clinodactyly	–	33	33	33.67	[4, 6, 8, 24, 26, 28, 32–34, 38, 43, 44, 50, 53, 54, 64, 68, 69, 71]
Brachymesophalangy	–	32	32	32.65	[7, 8, 25, 32, 38, 42, 44, 46, 50, 52, 66, 70–72]
Dysmorphic phalanx	–	5	5	5.10	[7, 38, 52]
Abnormal palmar crease on fingers	–	5	5	5.10	[4, 25, 26, 43, 72]
Arachnodactyly	–	6	6	6.12	[41–43, 62, 65, 66]
Small hands	–	12	12	12.24	[7, 8, 17, 18, 24, 32, 36, 44, 54, 71]
Single sandal gap	–	1	1	1.02	[30
Disproportionate growth of limbs	–	8	8	8.16	[28, 42, 43, 55, 61, 65, 69]
Urogenital					
Uterine hypoplasia	n/a	5	5	5.10	[52]
Ovarian hypoplasia	n/a	6	6	6.12	[52]
Small genitalia	n/a	6	6	6.12	[25, 31, 47, 65, 66]
General					
Hypotonia	–	10	10	10.20	[17, 23, 24, 26, 31, 59, 69, 75]
Growth retardation	+	74	75	76.53	[6–10, 15, 17, 18, 22–35, 37–40, 42–49, 51, 52, 54–56, 58–60, 64, 65, 67, 70–74, 76, 77]
Overweight	–	9	9	9.18	[30–32, 39, 47, 52, 54, 76]
Feeding problems	–	9	9	9.18	[31, 42, 54, 59, 69, 71]
Behavioral					
Retarded gross motor milestones	–	19	19	19.39	[7, 8, 18, 22, 29, 30, 32, 38, 40, 44, 47, 48, 55, 59, 60, 69, 73, 77]
Developmental delay	–	27	27	27.55	[4, 6, 17, 18, 22, 24, 27, 28, 30, 32, 40, 47, 48, 54–56, 59, 60, 63, 72, 73, 77]
Mental deficit	–	40	40	40.82	[4, 7–10, 15, 26–29, 31, 32, 35, 38, 39, 41, 44, 46, 47, 49, 54, 59, 64, 71, 72, 75, 77]
Visual discrimination	–	1	1	1.02	[44]
Language deficit	–	27	27	27.55	[4, 15, 18, 22, 23, 27, 31, 32, 35, 38, 44, 48, 51, 53–55, 59, 63, 65, 69, 73, 75, 76]
Aggressive mood	–	5	5	5.10	[23, 35, 69, 70]

*n/a* not applicable

## Discussion

Ring chromosome 15 syndrome is a rare genetic disorder, which is still not completely understood. Ring chromosomes are formed due to a loss of the distal fraction of the chromosome, followed by a fusion event to the terminal region of the short arm of the same chromosome [1, 5]. The size and position of the lost fragment determines the phenotype of the patients. As the nature of the chromosome is unstable and depending on what genes have been lost, the spectrum of clinical features can vary from patient to patient [1, 4].

Copy number variations (CNVs) are one of the most referred causes of human disease pathogenesis because they often include important and functional DNA sequences. It has been reported that gene expression variability is highly related to CNVs larger than 40 kb, which is consistent with the number of copies lost in this study [13]. Geneticists are no longer associating diseases with common genetic variants inherited through different generations, but with large and rare structural variants of recent origin (like ring formations). Large and rare structural variants of recent origin are responsible for serious conditions like autism, schizophrenia, and intellectual disability; intellectual disability is one of the most common phenotypes found in the different clinical cases analyzed [14].

The clinical manifestations of the case described in this study fit well with previous ones reported in literature. In most of the cases, there is the presence of invariable size rings with a minimal loss of genetic material. We identified that there is a common breakpoint in most: the genomic region 15q26. Duplication, deletions, and gains in this region could be associated with different phenotypic manifestations as the region harbors different genes [15]. For instance, insulin-like growth factor 1 receptor (*IGF1R*) is involved in normal growth and development [16]. Mutations and changes in this gene, such as abnormal number of structures of receptor, can lead to different pathologies associated with growth deficit, such as Prader–Willi syndrome and Silver–Russell syndrome [17, 18]. The loss of one copy of the gene can also lead to abnormal head and body size. Other genes such as synemin (*SYNM*) and tetratricopeptide repeat domain 23 (*TTC23*)*,* which play a role in the assembly of intermediate filaments in the Z-ring, are shared among previously described cases. Abnormalities in these genes can lead to musculoskeletal disorders, such as delayed bone age, digit and finger clinodactyly, and brachydactyly [19].

In addition, with the array analysis we found two other chromosomes involved in loss and gain of genetic material: chromosome 14 and chromosome X. Genes identified to be part of the region lost, Xq26.3, included several members of the family cancer/testis antigen family 45 (*CT45*), such as *CT45A1*–*CT45A6* associated with oncogenic function [20]. On the other hand, gain of genetic material on region Xq11.1 involved genes *SPIN4* and *LOC92249*; losses and mutations on these genes have been associated with periodontitis phenotype [21].

**Fig. 2:**
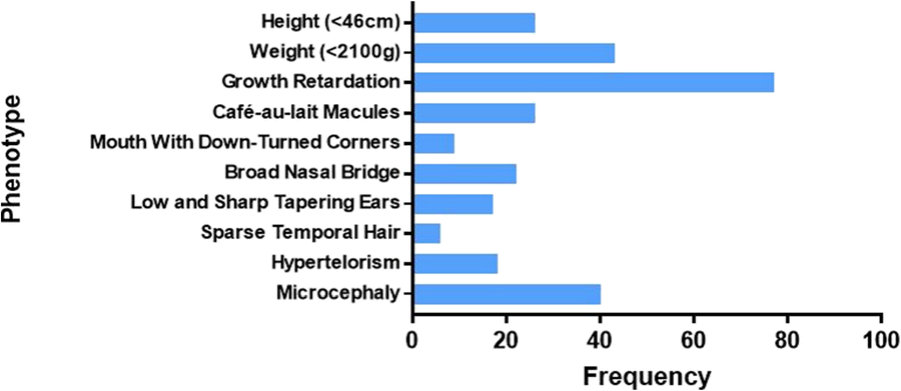
Shared phenotype frequencies among 97 clinical cases and the present case

**Fig. 3:**
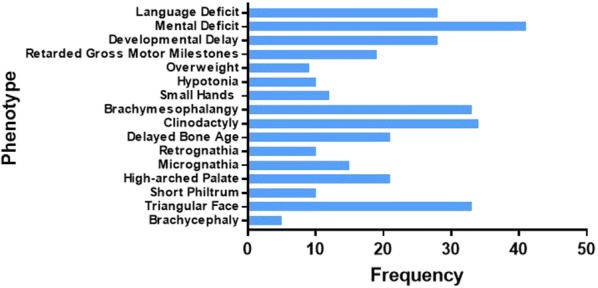
Shared phenotype frequencies among the 97 reviewed clinical cases

**Fig. 4:**
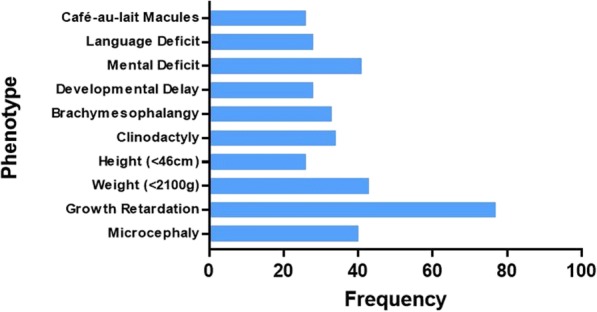
Frequencies of the most common phenotype expressed by 97 clinical cases and the patient of this study

The literature review for phenotype comparison (Table 3) also led to interesting findings; the different phenotypes of all 98 reviewed cases were categorized. We reported the frequency of shared phenotypes and found that growth retardation, microcephaly, and a low weight were the main characteristics in cases with r(15) (Fig. 2). Figure 3 also shows phenotypes shared among the clinical cases in the literature with mental deficit, brachymesophalangy, clinodactyly, and triangular face as the dominant traits. Finally, the most shared phenotypes between all the cases were selected and compared (Fig. 4) with the aim of finding a common trait among the patients; we found that growth retardation was present in almost 80% of the cases. However, due to the low statistical significance of the frequency of the phenotype expressed by all cases, a common phenotype for patients with ring chromosome 15 could not be established and is highly restricted to the region of chromosome 15 lost due to the ring formation. It has been suggested that the clinical phenotype of patients with ring chromosome depends on two main features: the first is related to telomeric deletions responsible for the ring formation, and the second is directly related to the amount of DNA lost and the functional genes involved in this region [5].

## Conclusions

Ring chromosome 15 syndrome shows a heterogeneous phenotype which is dependent on the region and the genes involved in the break, although there are some traits like the ones shown in Figs. 2 and 3 that have more than 40% frequency in the individual cases reviewed. Those traits can be suspected to be the shared phenotype of r(15) syndrome. Cases that show a breakpoint on genomic region 15q26 show common dysmorphic features, such as musculoskeletal abnormalities and growth retardation. Furthermore, cytogenetic and molecular approaches could facilitate the association of phenotypic and genotypic correlations. Further investigation and description of patients’ cases could provide an insight to the genetic aberrations involved in ring chromosome 15 disorders, in order to offer more information on the genes and the genomic regions affected.

## Abbreviations

APGAR: Appearance, Pulse, Grimace, Activity, and Respiration
CNV: Copy number variation
*CT45*: Cancer/testis antigen family 45
GTG: Giemsa trypsin banding
HC: Head circumference
*IGF1R*: Insulin-like growth factor 1 receptor
r(15): Ring 15
*SPIN4*: Spindlin family member 4
*SYNM*: Synemin
*TTC23*: Tetratricopeptide repeat domain 23
